# Genetic Tools for the Analysis of *Drosophila* Stomatogastric Nervous System Development

**DOI:** 10.1371/journal.pone.0128290

**Published:** 2015-06-08

**Authors:** Karla Hernández, Logan G. Myers, Micah Bowser, Thomas Kidd

**Affiliations:** Biology/MS 314, University of Nevada, Reno, Nevada, United States of America; University of Würzburg, GERMANY

## Abstract

The *Drosophila* stomatogastric nervous system (SNS) is a compact collection of neurons that arises from the migration of neural precursors. Here we describe genetic tools allowing functional analysis of the SNS during the migratory phase of development. We constructed GAL4 lines driven by fragments of the *Ret* promoter, which yielded expression in a subset of migrating neural SNS precursors and also included a distinct set of midgut associated cells. Screening of additional GAL4 lines driven by fragments of the *Gfrl/Munin*, *forkhead*, *twist* and *goosecoid (Gsc)* promoters identified a *Gsc* fragment with expression from initial selection of SNS precursors until the end of embryogenesis. Inhibition of *EGFR* signaling using three identified lines disrupted the correct patterning of the frontal and recurrent nerves. To manipulate the environment traveled by SNS precursors, a *FasII-GAL4* line with strong expression throughout the entire intestinal tract was identified. The transgenic lines described offer the ability to specifically manipulate the migration of SNS precursors and will allow the modeling and in-depth analysis of neuronal migration in ENS disorders such as Hirschsprung’s disease.

## Introduction

The invertebrate stomatogastric nervous system (SNS) has provided a wealth of information on the functioning of simple neural networks [1]. In *Drosophila*, all aspects of the adult gut including the enteric nervous system (ENS) have received intense attention in recent years [2]. After initial characterization of the embryonic development of the SNS primarily by the Hartenstein and Jäckle groups [3–9], the early SNS has received relatively little consideration. This is surprising as the SNS is a simple developmental system and likely to be of clinical relevance to vertebrate ENS disorders.

The SNS begins as three epithelial pouches in the primitive mouth (stomatogastric) that delaminate and migrate along the developing foregut as coherent clusters (referred to as invaginating SNS precursors or iSNSPs; reviewed in [4]). An additional group of cells (dSNSPs) delaminate in front of the iSNSPs [5]. The SNS anlage is located within the roof epithelium of the stomodeum, the primitive mouth of the embryo. Within the anlage, three single cells, called tip cells (tSNSPs), are selected by the action of the proneural (*achate-scute*), neurogenic (*Notch*) and wingless (*wg*) genes [8]. The tip cells secrete an Epidermal Growth Factor (EGF), Spitz, which induces EGF receptor (EGFR) signaling in the surrounding cells, inducing them to delaminate from the epithelium and form migratory vesicles [3, 9]. These three clusters of cells migrate along the foregut and then start to produce daughter cells that separate and migrate both anteriorly and posteriorly to form discrete ganglia [5]. In anterior to posterior order, the ganglia are: the frontal ganglion which lies on top of the pharynx anterior to the brain commissure, two sets of esophageal ganglia which lie alongside the esophagus, and the proventricular ganglion which innervates the crop-like proventriculus that forms at the junction of the foregut and midgut [10]. Cells from each iSNSP cluster contribute to each of the ganglia, whereas the dSNSPs contribute only to the frontal ganglion [5].

**Fig 1:**
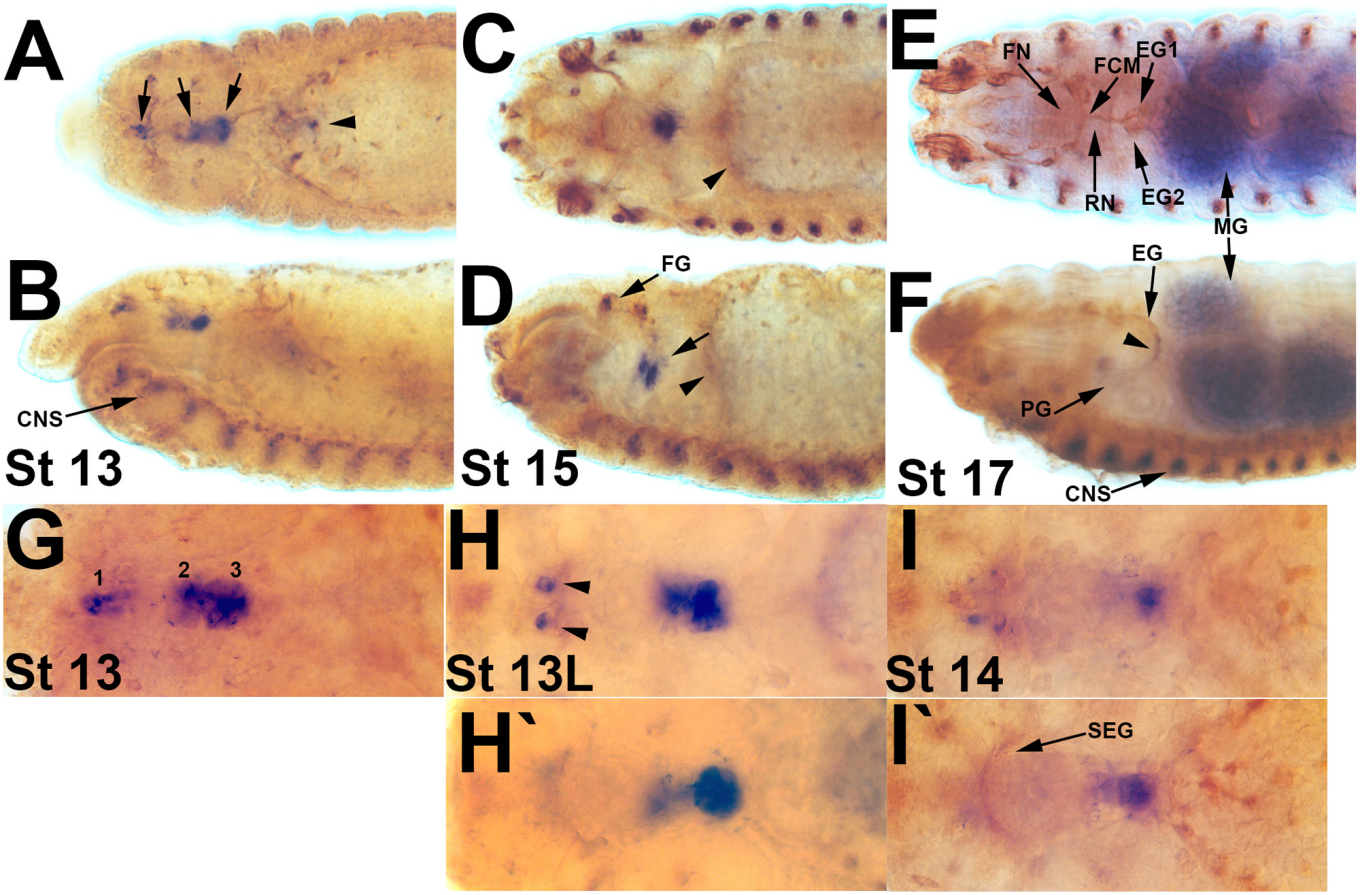
*Ret* expression in the developing SNS. *Drosophila* embryos with an in situ hybridization for the *Ret* gene (dark blue) and antibody staining with the 22c10 antibody (brown) to reveal the SNS, the PNS and elements of the CNS. A, C, E, G, H and I are dorsal views, B, D and F are lateral views. (**A, B**) Stage 13 embryo with expression in the migrating SNS clusters (arrows). Limited expression can also be seen in a discrete set of cells of the anterior midgut (arrowhead) and in the CNS midline at the bottom of panel B (CNS). (**C, D**) Stage 15 embryo in which the esophagus has started to loop. The three SNS clusters are immediately adjacent to one another within the loop and all express *Ret* (arrow). Additional *Ret* staining occurs in the developing frontal ganglion (FG). Faint expression can be seen in the anterior midgut (arrowheads), the ventral midline and PNS cells towards the anterior of the embryo. (**E, F**) Stage 17 embryo with *Ret* expression in some cells of the esophageal ganglion (EG) and proventricular ganglion (PG). Significant *Ret* expression is observed in the midgut (MG) and the CNS midline (CNS). (**G**) Expression of *Ret* in the three migrating SNS clusters in a stage 13 embryo. (**H, H’**) Two different focal planes of late stage 13 embryo. Ret is expressed in the SNS clusters which are clustered in the looping esophagus (compare to D), and in CNS cells that project through the subesophageal ganglion (arrows). (**I, I’**) Stage 14 embryo with diminishing *Ret* expression. Some axons of the subesophageal ganglion (SEG) are labeled by 22c10.

The fly SNS has strong parallels with the vertebrate neural crest as epithelial cells delaminate and migrate to their final destinations. In vertebrates, the *RET* receptor tyrosine kinase has a critical role in the migration of enteric neuron precursors and mutations are a key cause of Hirschsprung’s disease in which the colon and rectum have severely decreased innervation [11–13]. Intriguingly the fly *Ret* gene is expressed in the migrating SNS precursors (Fig 1) suggesting there may be a shared evolutionary origin [14]. *Drosophila Ret* mutants affect dendrite growth but have not yet been examined for SNS defects [15]. We wished to generate transgenic reagents specific to the developing SNS as many developmental genes affect multiple stages and tissues during development, which can hinder phenotypic analysis. Some of the reagents may allow functional assays of feeding and peristalsis to be conducted in larvae. We constructed fragments of the *Ret* promoter to the GAL4 gene and also screened additional GAL4 lines. Three specific GAL4 lines, *GscG-GAL4*, *Gfrla-GAL4* and *RetP-GAL4*, were identified that allow the manipulation of SNS precursors and these will be made available to the research community.

## Materials and Methods

### Molecular Biology

**Fig 2:**
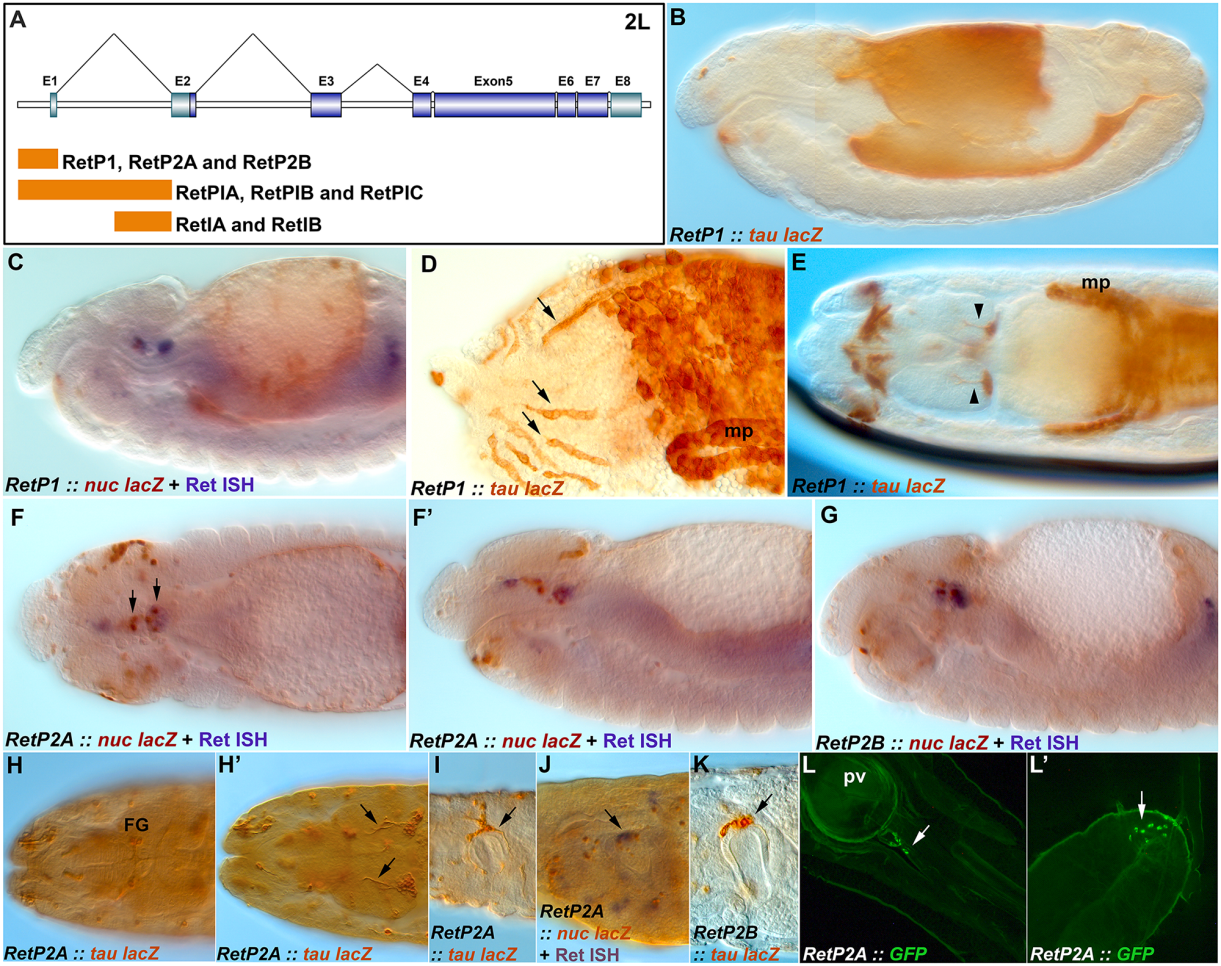
Expression of Ret-GAL4 transgenes. Embryos and larvae with Ret-GAL4 lines driving expression of either nuclear lacZ (*nuc-lacZ*) or tau-lacZ reporters (both brown) with select counterstaining with an in situ hybridization probe for *Ret* (blue). (**A**) Diagram of the *Drosophila Ret* gene showing the location of fragments used to construct GAL4 lines. (**B**) Expression pattern of *RetP1-GAL4* driving *tau-lacZ* showing broad expression throughout the inner lining of the gut. (**C**) *RetP1-GAL4* driving expression of *nuc-lacZ* showing expression in discrete gut cells. (**D**) *RetP1-GAL4* driving expression of *tau-lacZ* with individual cells in the midgut sometimes aligning into linear arrays (arrows). (**E**) *RetP1-GAL4* and *tau-lacZ* with expression in brain neurons (arrowheads), elements of the CNS and PNS at the anterior of the embryo (left), malphigian tubules (mp) and hindgut. (**F**) Dorsal view of an embryo with *RetP2-GAL4* driving *nuc-lacZ* in a subset of migrating SNS precursors (arrows and blue stain) and the gut. (**F’**) Lateral view of the same embryo as F with SNS precursors and gut staining visible. (**G**) An independently recovered line of *RetP2* showing similar staining as F. (**H, H’**) Stage 17 embryo showing persistence of RetP2 expression in the neurons of the frontal ganglion (FG) and brain neurons (arrows). (**I**) First instar larvae with *RetP2* and *tau-lacZ* displaying prominent staining in the proventricular ganglion (arrow). (**J**) Late stage 17 embryo showing overlap of *RetP2*, *nuc-lacZ* and *Ret* mRNA expression in the proventricular ganglion(arrow). (**K**) First instar larva with RetP2-GAL4 and tau-lacZ expression on the proventricular ganglion (arrow). (**L,L’**) Expression of *UAS-CD8-GFP* under control of RetP2A-GAL4 in a second instar larva showing expression (arrows) in cells adjacent to and downstream of the proventriculus (pv).

A 527 base pair fragment upstream of the Ret transcription start site was amplified with Phusion high fidelity DNA polymerase from genomic DNA derived from an Exelixis isogenic stock [16] with CCAGGTAAACCCTTTTATCG (forward) and CCGCGGAAATACTTTTTGG (reverse) primers (written from 5’ to 3’), cloned into pCR8/GW/TOPO (Life Technologies Inc.) and subcloned into the StuI and EcoRI sites of pPTGAL (Addgene; [17]). P-element injections were performed by Genetic Services, Inc. (Sudbury, MA) and one transformant was recovered. The same fragment was cloned into pENTR/D-TOPO (Life Technologies Inc.) and subcloned into pBPGUw (Addgene; [18] using LR Clonase II (Life Technologies Inc.). Two additional fragments were amplified (Fig 2) and cloned the same way using the forward primer above and GTATGACTGCTAATTATT (reverse), and GTCGTATGTTATTAGCAT and CGGATATTTAGACCACGAAC primers. Sequencing of constructs was performed by the Nevada Genomics Center. Injection using phiC31 integrase into the *attP2* landing site (Bloomington #25710 *nos-phiC31-int*.*NLS*, *attP2*) was performed by Rainbow Transgenics (Camarillo, CA) and Genetic Services Inc. Six additional transformants were recovered.

### Immunohistochemistry

Antibody staining was performed as described in [19], and in situ hybridizations per [20]. Ret probe was generated by transcription of a 3 kilobase genomic fragment cloned in pBluescript. We generally use 70% glycerol in PBS or 0.1M Tris pH 8.0 as clearing agents. However, the lipid rich midgut can be hard to resolve with microscopy, so we tested several clearing protocols including ClearT [21]. We found that the best results were obtained with either Focus Clear and Rapid Clear (Cedarlane; [22]); both reagents were also useful for imaging late stage 17 embryos.

### *Drosophila* Genetics

Janelia Farm GAL4 lines were all obtained through the Bloomington Drosophila Stock Center (BDSC). *Gsc*-GAL4 lines are: 46772, 48376, 46773, 48377, 40381, 40382, and 40383. *Gfrl*-GAL4 lines are: 47237, 47238, 47239, 47275, 40663, 40664, 40665, 40666 and 40667. *fkh*-GAL4 lines are: 47326, 48746, 48764, and 48795. *twi*-GAL4 lines are: 46150, 48725, 48729, and 48760. Please note some of these lines are no longer available but we are happy to supply them on request. The stock number for *FasII-GAL4* is 46123, *UAS-EGFR RNAi* on III is 36770 and *UAS-EGFR-DN* on II and III 5364. *UAS-nuclear-lacZ* and *UAS-CD8-GFP* were obtained from the BDSC. *UAS-tau-lacZ* was obtained from M. Fujioka. Several of the GAL4 lines are no longer available from Bloomington and we are more than willing to supply them upon request. The w^1118^ stock was the most reliable wild type stock as other reference stocks do not consistently display the wild type neuroanatomy described in previous publications.

### Statistics

For each genotype, stage 17 embryos were collected at random and scored for the presence, absence or thinning of the frontal nerve, and for defasciculation defects in the recurrent nerve. At least ten embryos were collected for each genotype. The 95% confidence interval and the Fisher exact test with two tails for the phenotypes was calculated using the GraphPad website (www.graphpad.com/quickcalcs). Statistical significance was assessed using the Bonferroni correction.

## Results

### *Ret* expression in the developing SNS

Expression of the *Ret* gene has been thoroughly documented in the *Drosophila* embryo [14]. We confirmed expression in the migrating SNS precursors (Fig 1A–1D and 1G–1I). *Ret* expression is dynamic, with expression reduced in SNS cells that have completed migration (Fig 1E and 1F). We also noted expression in the anterior midgut, which is present throughout the midgut by the end of embryogenesis (Fig 1E and 1F). Gut expression is robust but appears weaker than expression in other tissues. We additionally noted expression in a paired set of CNS neurons at the level of the subesophageal ganglia (Fig 1H) along with an ordered row of midline cells in the ventral nerve cord.

### Generation of *Ret-GAL4* lines

Traditional pan-neural promoters do not express during SNS precursor migration and previously identified promoter elements either have broad or highly limited expression [5, 9]. We chose to place fragments of the *Ret* promoter in front of the GAL4 gene with the goal of generating more SNS specific reagents. *Ret* is distinguished by a short promoter region upstream of the transcriptional start and three large introns (Fig 2A). We cloned the promoter region into the pPTGAL vector [17] and generated transformants using P element transposase (*RetP1-GAL4*). We also placed the promoter into the pBPGUw vector (*RetP2-GAL4*) [18], as well as the promoter fused to the first intron (*RetPI-GAL4*), and the second half of the first intron (*RetI-GAL4*; Fig 2A); transformants were generated using the PhiC31 integrase. Transformant recovery proved especially difficult for all transgenes and even identical constructs integrated into the same site yielded differences in expression (Table 1), suggesting there may be negative selective pressure towards the *Ret* control regions when fused to *GAL4*. The promoter constructs yielded broad expression particularly in the epithelial lining of the midgut (Fig 2B). Expression occurs after migration of the endodermal cells [5] and often appears continuous, but then becomes restricted to a large number of discrete cells, sometimes in linear arrangements (Fig 2C and 2D). Midgut expression is most pronounced in the *RetP1-GAL4* construct. Expression was also seen in the brain (Fig 2E), in what may be a single lineage for either the mushroom bodies or a more basal lateral cluster [23]. The pBPGUw insertions (*RetP2A*,*B-GAL4*) displayed expression in a subset of the migrating SNS precursors as defined by *Ret* expression (Fig 2F and 2G). Additional isolated cells throughout the head region express Ret as has been seen for *Ret* mRNA [14], including a subset of cells projecting through the subesophageal commissure. Expression of *Ret-P2* persists to the end of embryogenesis and was found to label a subset of cells in the frontal and esophageal ganglia (Fig 2H). Strong expression of reporters was observed in SNS cells at the proventriculus and additional labeled cells further along the gut (Fig 2I, 2K and 2L). Finally expression was also observed in larval midgut and body wall neurons (Fig 2L’). Additionally, we cloned the entire first intron into pBPGUw yielding strong expression in the midgut and hindgut, but not in the SNS (Table 1). The second half of the first intron produced expression in gut related tissues but not the SNS (Table 1). The *RetP2* transgene appeared the most useful for SNS manipulation even though expression was only observed in a subset of cells, because expression persists into larval stages primarily in the proventricular ganglion.

**Table 1:** Summary of GAL4 line embryonic expression patterns.

Gal 4 Driver Line	Expression Time	Expression Place	Primary Expression Feature	Embryonic SNS expression (stage)	Larval Expression (mCD8 GFP)
Gsc A 46772	embryo 11–17	Surrounding brain lobes and weak midline (15–17)	Exterior brain lobes	-	-
Gsc B 48376	embryo 11–17	Broad brain lobe expression (11–17); anterior sensory neurons (11–17); lining of esophagus (13–16)	Full brain lobes	-	-
Gsc C 46773	embryo 13–17	Weak CNS expression (11–17)	Weak CNS	-	-
Gsc D 48377	embryo 12–17	Strong CNS expression (12–17)	Complete CNS	-	-
Gsc E 40381	embryo 11–17	Weak CNS expression (12–17)	Weak CNS	-	-
Gsc F 40382	embryo 12–16	Esophagus and foregut/esophageal ganglion (12–16); mild outer brain lobe (12–16); small hindgut segment (12–16)	SNS	st 12–16	Esophagus (pharyngeal muscles?); hindgut
Gsc G 40383	embryo 11–17	Pre-migrating SNS (11); early esophagus and foregut (11–13); proventriculus/foregut (16/17); frontal ganglion and FNJ (17); posterior brain lobe cluster (15–17)	Foregut/SNS	st 11–17	-
Mun α 47237	embryo 10–17	Brain lobes (12–17); SNS precursors (10/11); anterior end of midline (12–16)	Very early SNS	st 11–14	Anterior midgut cell bodies; hindgut
Mun β 47238	embryo 11–17	Anterior tip of esophagus (13–15); esophageal ganglion (13); brain lobes (15–17); large posterior brain lobe cluster (16–17); anterior receptor cell clusters (11–17)	Mid-stages SNS marker	st 11–13	Anterior midgut cell bodies
Mun γ 47239	embryo 11–17	Lining developing esophagus (12–16); optic lobe precursors (11–12); weak brain lobe (15–17); receptor cells in anterior end of embryo (16–17)	Early SNS/anterior sensory neurons	st 11–15	-
Mun δ 47275	embryo 11, 15–17	SNS precursors (11); anterior sensory receptors (16–17)	Mild anterior sensory neurons	st 11	-
Mun ε 40663	embryo 13–17	Dorsal closure (13–17)	Dorsal vessel	-	-
Mun Ζ 40664	embryo 11–17	Midline precursors (13–17); presumptive foregut/hindgut (9–11); lining of developing esophagus (13–15); developing brain lobes (13–17)	Developing CNS	st 13	Similar expression as Mun I
Mun Η 40665	embryo 13–17	Broad expression in small punctate 13–17	Nonspecific	-	-
Mun Θ 40666	embryo 11–17	Pre-migrating SNS and early esophagus (11); sporadic and nonspecific esophageal tissue (16–17); receptor cells in anterior end of embryo (13–17)	Anterior dorsal sensory neurons	st 11	None observed
Mun Ι 40667	embryo 11–17	Presumptive hindgut (11); spread along developing esophagus (13–17); weak midline glia expression (13–17); brain lobes (14–17)	Early foregut/hindgut; midline glia	st 15–17	Posterior to PV; anterior midgut cell bodies; midbrain?; IMR?
Fkh 1 47326	embryo 11–17	Hindgut lining (11–17); light expression in whole midgut (11–17)	Hindgut lining	-	Hindgut
Fkh 2 48746	embryo 11–17	Weak CNS expression (11–17)	Non specific	-	-
Fkh 3 48764	embryo 9–17	Foregut/hindgut (9–12); complete Intestinal tract (13–17); Malpighian tubules (13–16); mild CNS expression (13–17)	CNS/Intestinal Anatomy outline	-	-
Fkh 4 48795	embryo 11–17	Similar to Fkh 3; intestinal tract expression (11–17); Malpighian tubules (15/16)	Intestinal anatomy outline	st 11–15	-
Twi 1 46150	embryo 13–17	SNS cluster (13); esophageal/pharyngeal muscles, dorsal side of esophagus/EG? (15–17)	Esophageal/pharyngeal clusters	st 13–17	Anterior sensory neurons
Twi 2 48725	embryo 13–17	Mild CNS expression (13–17)	Weak CNS	-	-
Twi 3 48729	embryo 11–17	Developing anterior Sensory receptors (11–17); mild CNS (13–17);	Anterior sensory neurons	-	-
Twi 4 48760	embryo 11–17	Developing CNS (11–17)	CNS	st 13,17	-
RetP1 (Herna#1 in pPTGAL)	embryo 12–17	Distinct expression in cells of the dorsal fold (14); putative adult midgut precursors and other endoderm/endoderm adjacent cells (14–17); specific subset of brain cells (15–16); periventricular ganglion, dorsal pharyngeal muscles, Malpighian tubules (17)	CNS; SNS; foregut/midgut/hindgut; brain lobes	st 13–17	Brain; posterior to PV; anterior midgut cell bodies and hindgut
RetP2A (Herna#2 in pBPGUw)	embryo 12–17	Pre-migrating SNS clusters (11–12); migrating SNS clusters (13–16); specific subset of brain cells, putative adult midgut precursors and other endoderm/endoderm adjacent cells and Malpighian tubules (15–17)	CNS; SNS subset; foregut/midgut/hindgut; brain lobes	st 12–17	SNS; posterior to PV; anterior midgut cell bodies
RetP2B (Herna#3 pBPGUw)	embryo 12–17	Distinct expression in cells in the esophageal clusters (13–14); esophageal and periventricular ganglion, specific subset of brain cells, putative adult midgut precursors and other endoderm/endoderm adjacent cells (15–17)	CNS; SNS subset; foregut/midgut/hindgut; brain lobes	st 12–17	SNS; posterior to PV; anterior midgut cell bodies
RetPIA (Herna#4 pBPGUw)	embryo 13–17	Tracheal/peripheral (ventral) expression (13–14); midgut lining (endoderm/endoderm adjacent cells) (14–17 and later)	PNS (ventral); midgut	-	Midgut and hindgut
RetPIB (Herna#5 pBPGUw)	embryo 13–17	Proventriculus (13–14); minimal midgut/hindgut lining (endoderm/endoderm adjacent cells) (15–17); cephalopharyngeal ganglia/pharyngeal muscles (17)	Proventriculus; midgut; hindgut lining	st 13–14 (Proventriculus)	-
RetPIC (Herna#6 pBPGUw)	embryo 12–17	(Anterior) midgut lining (endoderm/endoderm adjacent cells) (14–17); hindgut lining (16–17)	Anterior midgut; hindgut	-	Midgut and hindgut
RetIA (Herna#7) pBPGUw)	embryo 12–17	CNS, broad PNS expression, trachea (12–16); proventriculus (16–17)	CNS/PNS; proventriculus	st 16–17 (Proventriculus)	-
RetIB (Herna#8 pBPGUw)	embryo 11–17	Developing CNS (11); distinct expression in cells in the esophagus (12–17); proventriculus (16–17); (anterior) midgut lining (endoderm/endoderm adjacent cells) (15–17)	Proventriculus; anterior midgut	st 12–17 (Proventriculus)	-

### Identification of Additional SNS Specific GAL4 Lines

**Fig 3:**
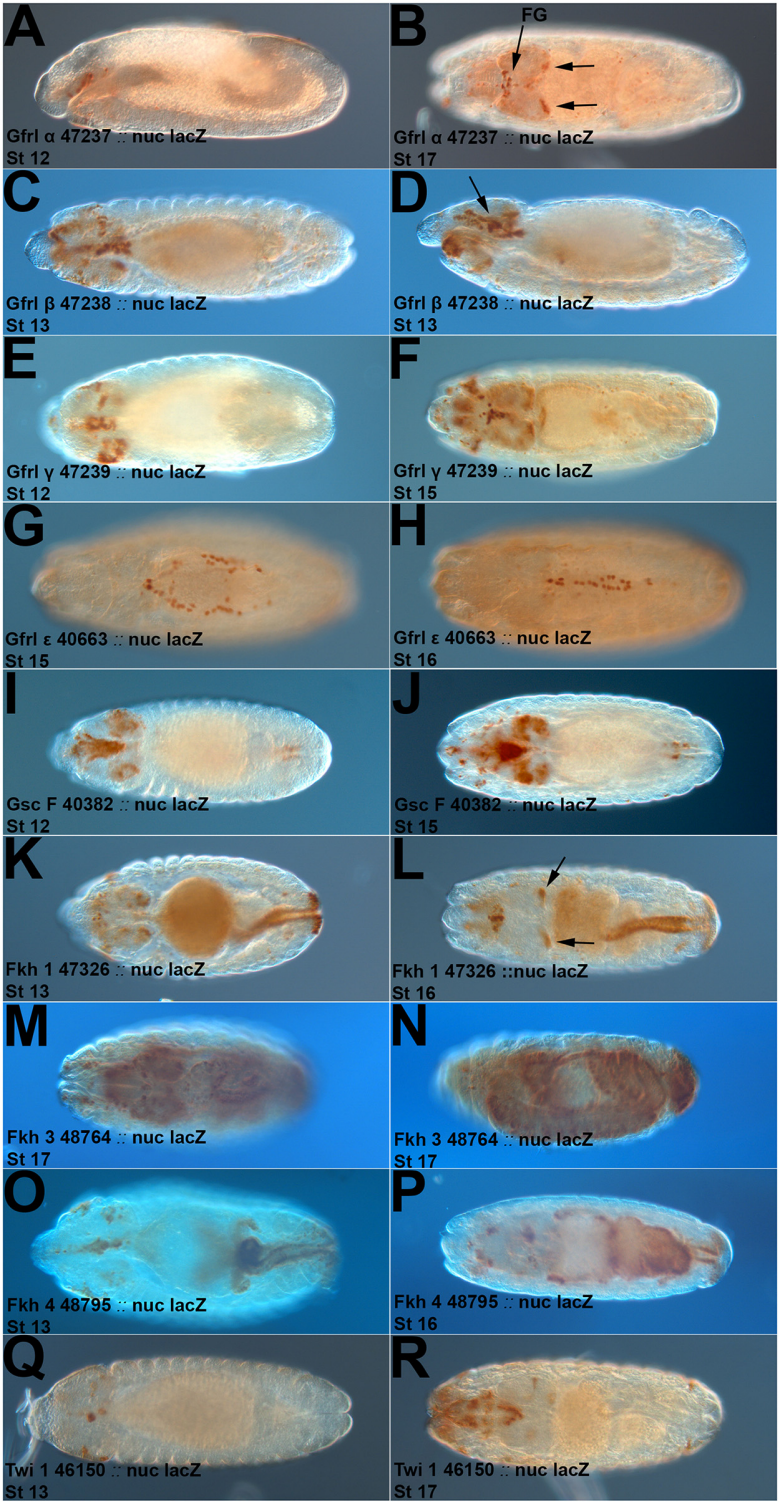
Expression of *Gfrl*, *Gsc* and *Fkh* GAL4 lines. Expression of *nuc-lacZ* or *tau-lacZ* (brown) by selected Janelia Farm GAL4 lines. The driver, reporter and embryo stage are noted on the Fig panel. Please also refer to Table 1. (**A**) *Gfrl* fragment driving expression in the roof of the stomatodeum in presumptive SNS precursor clusters and a few additional cells. (**B**) *Gfrl* fragment with expression in the frontal ganglion (FG) and brain lobe clusters (arrows). (**C, D**) Dorsal and lateral views of a *Gfrl* fragment driving expression in esophageal and SNS cells (arrow), cells presumed to be the subesophageal ganglion and additional cells. (**E**) Esophageal, SNS and brain lobe expression of a *Gfrl* fragment. (**F**) Esophageal, SNS and brain lobe expression with additional CNS and PNS cells. (**G, H**) Expression in cells of the leading edge during dorsal closure. (**I, J**) Expression of a *Gsc-GAL4* line in foregut, esophageal, SNS and brain cells. (**K, L**) Expression of a *Fkh-GAL4* line in the midgut, hindgut, brain lobe cells (arrows) and additional cells near the anterior of the embryo (left). (**M, N**) Expression of a *Fkh-GAL4* line throughout the gut and CNS in a late stage embryo. (**O, P**) *Fkh-GAL4* expression in the esophagus, SNS, brain lobes and gut cells in a pattern that resembles *RetP1*. (**Q**) *Twi-GAL4* expression in a subset of SNS cells. (**R**) Twi-GAL4 expression in the pharynx and additional unidentified cells that likely include parts of the SNS and PNS.

The limited SNS expression and additional expression of the *Ret* promoter fragments prompted us to look for additional reagents. We examined the Janelia Farm Fly Light GAL4 lines [18, 24] for driver fragments derived from genes with known SNS expression. Ret has an evolutionarily conserved co-receptor known as Gfrl or Munin in flies [25], and we tested nine *Gfrl* lines for SNS expression (Table 1). One line (#47237) had highly specific SNS expression from initial delamination of the SNS precursors until the end of embryogenesis (Fig 3A and 3B). This line (which we will refer to as *Gfrl-GAL4*) also displays brain lobe expression strongly resembling that of the RetP1 construct. Two additional lines (#47238, #47239) had broader expression in the esophagus and likely the SNS too (Fig 3C–3F). #47239 also has brain lobe expression. A fourth line expresses in cells at the leading edge of dorsal closure (Fig 3G and 3H). These lines have expression that appears significantly more restricted to the SNS than the Ret lines, with *Gfrla-GAL4* having the greatest potential for SNS manipulation. We also tested fragments of the *forkhead* (*fkh*), *Goosecoid* (*Gsc*) and *twist* (*twi*) genes as their expression has been reported in the SNS [8, 14, 26, 27]. Two *Gsc* lines were of interest, #40382 with esophageal and likely SNS expression (Fig 3I and 3J) and #40383 with strong specific SNS expression (see below). Three *fkh* lines had midgut and hindgut expression (Fig 3K–3P), with #47326 having brain lobe expression like Ret-P1, #48764 having very broad expression and #48795 strongly resembling Ret-P1 in the overall expression pattern. One *twi* line #46150 has potential expression in a very small subset of the SNS. However *Gsc* line #40383 stood out for its striking SNS specificity and duration of expression so we chose to characterize it further.

### Maternal Effect of the attP2 Integration Site Chromosome

The Fly Light lines are integrated into a third chromosome site, attP2, using the phiC31 site-specific integrase system [28]. We noticed that several GAL4 lines, but especially *GscG-GAL4* produced shorter embryos. This effect was regardless of reporter used and was only observed when the *GscG-GAL4* was the mother. The embryos themselves appear completely normal when assessed with 22c10 or 1D4 staining, just compressed along the anterior-posterior axis. A large number of embryos fail to hatch and the lines were quite difficult to maintain as homozygotes. Balancing in combination with a dominant male sterile mutation helped significantly. For all crosses, we used the GAL4 line as the male parent.

### Characterization of a *Gsc-GAL4* line

**Fig 4:**
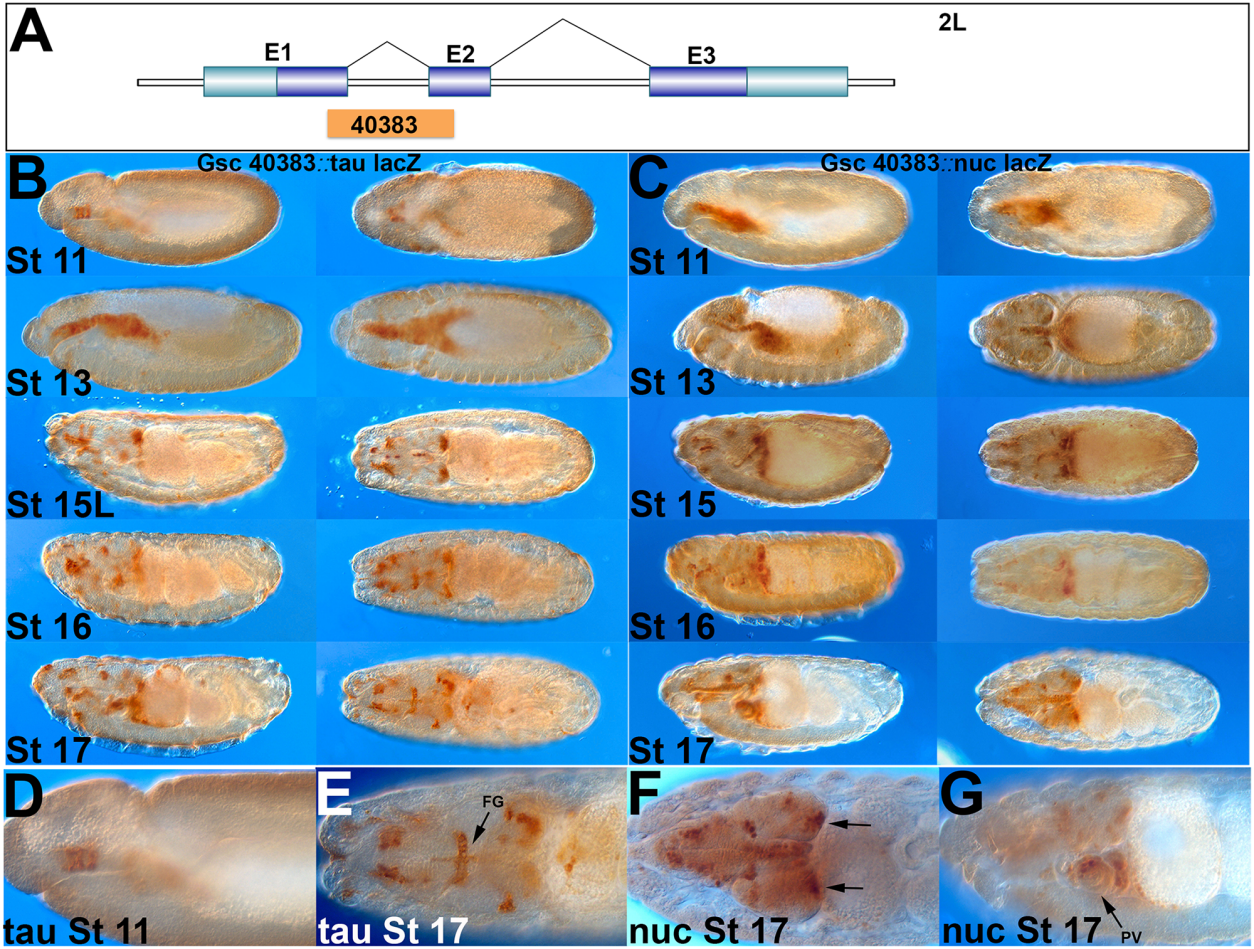
Characterization of the *GscG-GAL4* line. Developmental series of embryos expressing *tau-lacZ* (left hand columns) or *nuc-lacZ* (right hand columns) under control of the *GscG-GAL4* line. (**A**) Schematic of the position of the promoter fragment of *GscG-GAL4* inside the *Gsc* gene containing parts of exon 1 and 2. (**B**) Nuclear lacZ reporter displaying expression of *GscG-GAL4* in the initial SNS precursor clusters in the stomatodeum (stage 11, see also panel D), expanding to include most esophageal cells (stage 13), and resolving into SNS, brain lobe, subesophageal ganglion and PNS specific staining in later stages (15–17; see also panels E-G). (**C**) Tau-lacZ reporter in a similar developmental series to panel B. All SNS cells are labeled by the end of embryogenesis including the frontal ganglion, esophageal ganglia and proventriculus. (**D-E**) Higher magnification views showing the initial three delaminating SNS clusters at stage 11 and the entire frontal ganglion and nerve at stage 17 (FG). The nuclear staining shows the brain lobe clusters (arrows) as well as the proventriculus (PV).

A transposable element, SNS1-GAL4, inserted in the *Goosecoid* (*Gsc*) gene had been previously used to drive early SNS expression [9], which led us to test *Gsc* promoter fragments. *Gsc* #40383 expresses in all three SNS clusters from the start of SNS delamination until the end of embryogenesis (we will refer to this line as *GscG-GAL4*). This element contains parts of exon coding regions from the first and second exons of *Gsc* (Fig 4A). Early stage 11 expression of *GscG-GAL4* may be restricted to the three delaminating clusters but rapidly broadens to include cells in the underlying esophagus (Fig 4B and 4C). Expression in stage 16/17 is subsequently restricted to the migrating SNS precursors as well as the cluster of brain cells seen in *RetP1-GAL4* and the *Gfrl-GAL4* lines (Figs 2E, 3B,3F and 3L). Additional cells are seen in the head region as seen for *Ret* mRNA. At the end of embryogenesis the line has strong expression in the frontal nerve, frontal commissure, recurrent nerve, and proventricular ganglion (Fig 4E–4G). This line has the most complete expression in the anterior elements of the SNS, while *RetP1-GAL4* appears to express in more proventricular cells and for longer.

### Manipulation of SNS Migration using *Gfrl-GAL4* and *RetP2-GAL4*

**Fig 5:**
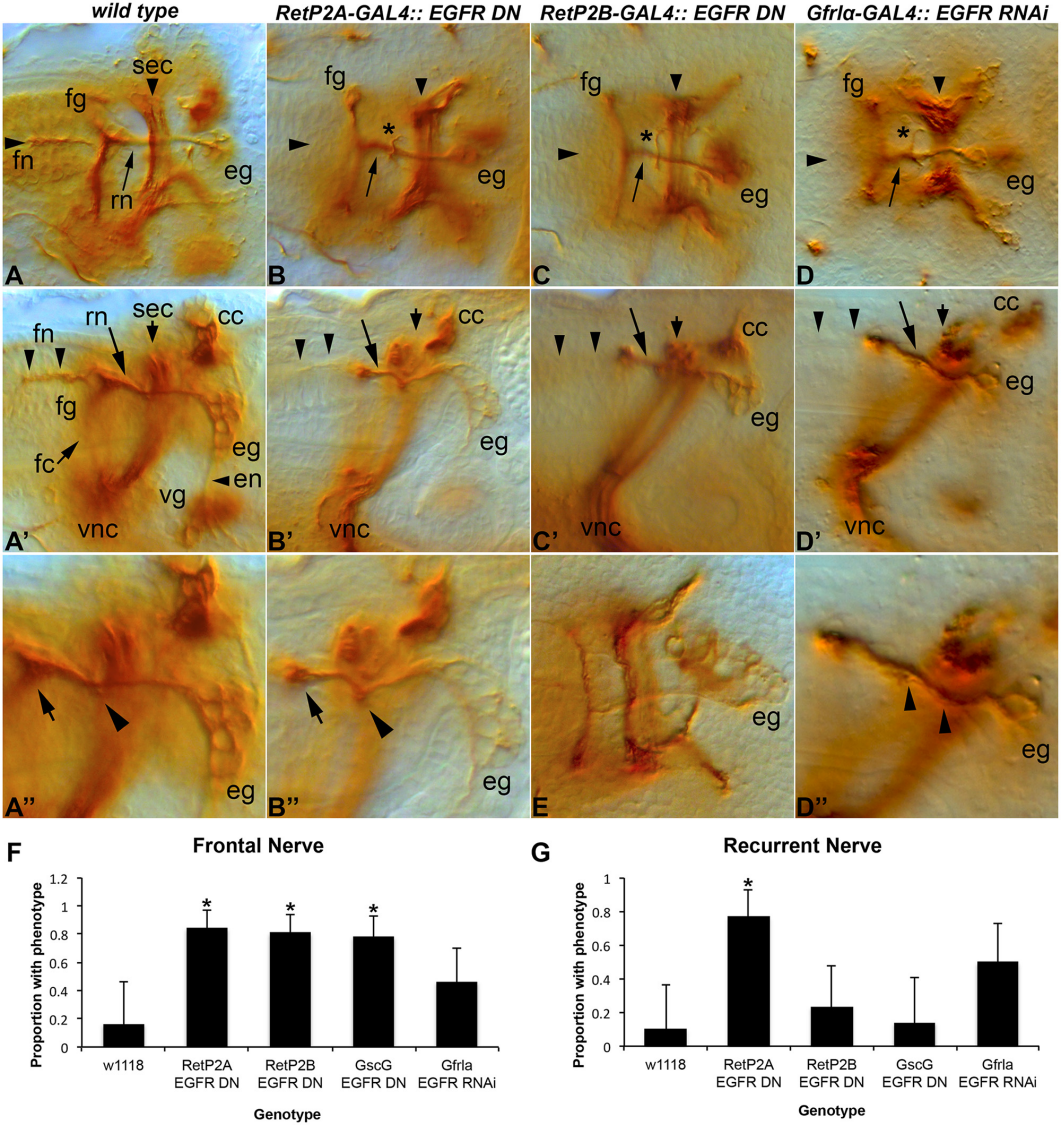
SNS manipulation using the *RetP2-GAL4* and *Gfrl-GAL4* lines. Anti-Fasciclin II staining (monoclonal antibody 1D4) revealing the mature embryonic SNS. We use 1D4 as it reliably stains the frontal nerve (fn) whereas 22c10 frequently fails to stain this nerve in wild type. (**A**) Dorsal view of a stage 17 wild type embryo with elements of the SNS labeled. The recurrent nerve (rn, arrow) runs from the esophageal ganglion (eg) along the esophagus underneath the supraesophageal commissure (sec, also known as the brain commissure) to the frontal ganglion (fg). The frontal nerve (fn, arrowhead) projects anteriorly from the frontal ganglion (fg). (A') Lateral view of the same embryo. The frontal connective (fc) which links the frontal ganglion to the brain, and the esophageal nerve (en) which links the esophageal ganglia to the ventricular ganglion (vg) can be seen. The corpora cardiaca (cc) neuroendocrine organ is visible close to the dorsal surface. (A'') Close-up of the frontal ganglion (arrow), recurrent nerve (arrowhead) and the esophageal ganglia (EG). A slight expansion of the recurrent nerve (arrowhead) can be seen, marking a location where defasciculated axons are often seen in transgenic manipulations. (**B**) *RetP2A-GAL4* driving a dominant negative *EGFR* transgen*e*. The frontal nerve (arrowhead) is absent and the recurrent nerve (arrow) exhibits a defasciculated axon or axons (asterisk). (B') Lateral view of the embryo in B. The frontal nerve is clearly missing (arrowheads). (B'') Close-up of the same embryo. The recurrent nerve displays a kink and expansion underneath the supraesophageal commissure (arrowhead). The frontal ganglion (arrow) is also visible. The number of cells in the esophageal ganglia appear comparable to wild type. (**C**) *RetP2B-GAL4* driving the dominant negative EGFR transgene. The frontal nerve is absent (arrowhead) and a defasciculated axon is crossing the recurrent nerve (asterisk). The latter axon may originate at the esophageal ganglia, but we have been unable to conclusively determine this for any examples studied. (C') Lateral view of the same embryo showing the absence of the frontal nerve (arrowheads) and axon defasciculation from the recurrent nerve (arrow). From this angle, at least one defasciculated axon appears to originate from the recurrent nerve itself. (**D**) *Gfrlα-GAL4* driving transgenic RNAi for *EGFR*. The frontal nerve is absent (arrowhead) and the recurrent nerve (arrow) is defasciculated (asterisk). (D') Lateral view of the same embryo showing the absence of the frontal nerve (arrowheads) and a slight swelling of the recurrent nerve (arrow) at the point that defasciculation occurs. (D'') Higher magnification view showing a defasciculated axon growing alongside the recurrent nerve from the esophageal ganglia to the point of defasciculation. (E) Late stage 17 embryo with the *EGFR* dominant negative transgene driven by *RetP2B-GAL4* showing that the number of cells within the esophageal ganglia appears unaffected by *EGFR* inhibition. (F, G) Quantification of frontal nerve defects (reduced or absent; F) or recurrent nerve (defasciculation; G) defects in the genotypes examined. The error bars represent the 95% confidence interval. Statistical significance (*) relative to the wild type control (w^1118^) was assessed using the Fisher exact test with two tails and the Bonferroni correction.

To test the utility of the identified GAL4 lines we chose to manipulate *EGFR* function. As noted above *EGFR* plays an important role in delamination of the SNS precursors and this early phenotype would preclude later phenotypes from being observed. We used *RetP2A- and RetP2B-GAL4* to drive an EGFR dominant negative construct [29, 30]. In each case we observed frequent loss or reduction of the frontal nerve and disruption of the recurrent nerve (Fig 5B, 5B', 5B'', 5C, 5C', 5E, 5F and 5G). We observed the same phenotype with *Gfrla-GAL4* driving *EGFR* RNAi (Fig 5D, 5D' and 5D''). Obtaining the same phenotype with three different GAL4 lines and two different constructs validates the lines identified. Similar results were obtained with *GscG-GAL4* and both dominant negative and RNAi transgenes. The observed phenotypes indicate that EGFR signaling is likely required for axon growth/guidance of the frontal and recurrent nerves. Based on the appearance of the esophageal ganglia (Fig B'', C'') cell number is not disrupted by *EGFR* inhibition. We could not accurately determine whether cells in the frontal ganglia are lost as anti-FasII staining does not reveal the cell bodies of the frontal ganglia in later stages (when the frontal nerve has developed). Most of the recurrent nerve axons project from the esophageal ganglia and project anteriorly, but a few appear to originate in the frontal ganglia and project posteriorly [10]. The recurrent nerve phenotype appears to arise from esophageal ganglion axons projecting anteriorly and defasciculating from the nerve (Fig 5D''). The axons can either change direction after passing under the brain commissure, or may fail to join the recurrent nerve in the first place projecting around the commissural surface of the brain lobe. Confocal microscopy could not further distinguish the origins of the phenotype (S1 Fig). Similar results were observed with *Gsc-*GAL4 and both *EGFR* RNAi and the dominant negative transgene (Fig 5F and 5G and S2 Fig). With our current level of analysis, we cannot rule out alternative explanations including non-cell autonomous effects on brain neurons although we think this unlikely. We note that similar recurrent nerve phenotypes were observed for the *misshapen*/*Ste20/l(3)6683* kinase that regulates MAPK signaling [7]. The *Gsc* expression pattern suggests there could be a cell type boundary at or near the defasciculation point at the pharynx-esophageal junction [26], perhaps indicating that secreted cues change at this position and that there could be choice point for growing axons.

### A GAL4 Line for Gut Specific Expression

**Fig 6:**
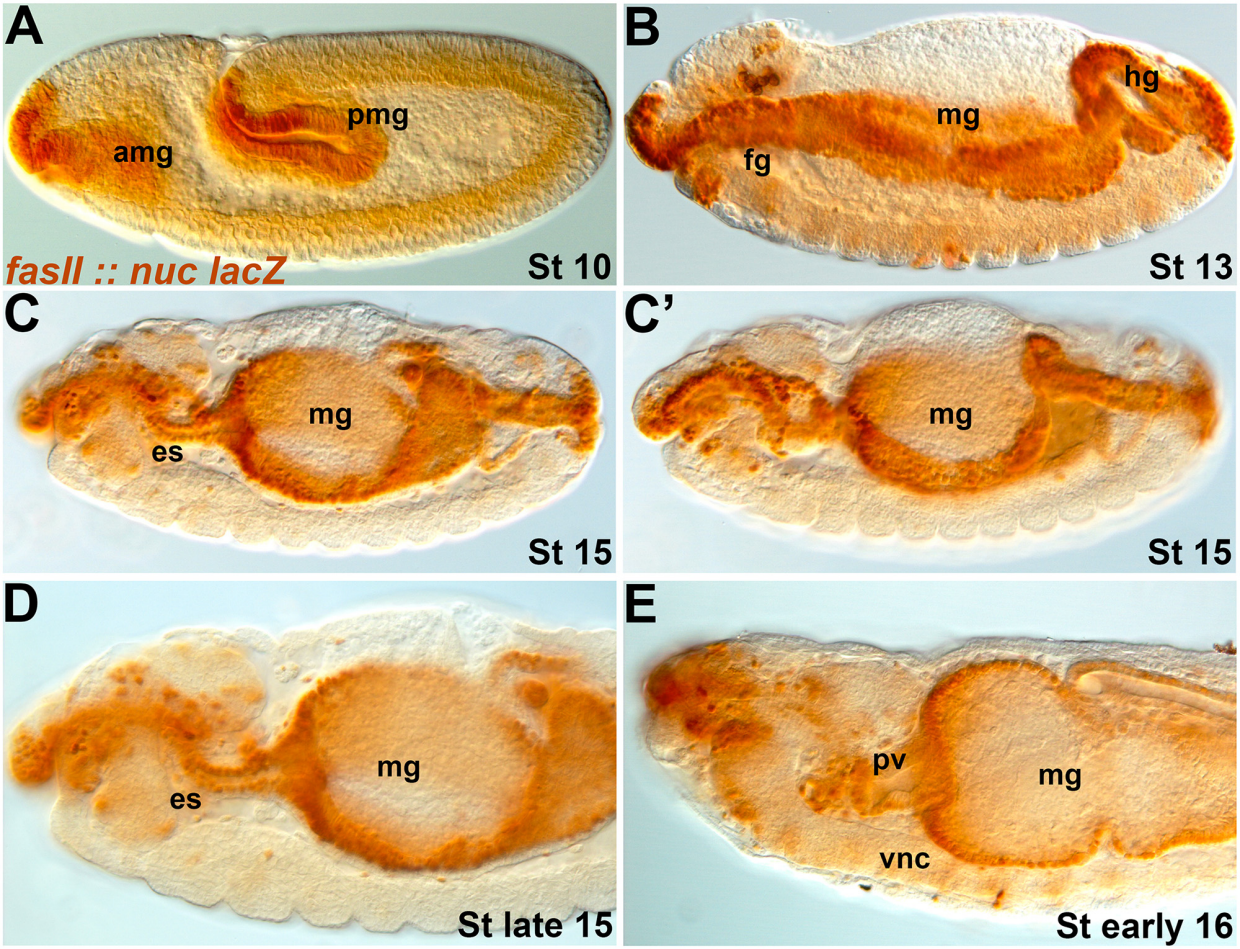
A FasII-GAL4 line drives in all parts of the developing gut. Lateral views of embryos with a nuclear lacZ reporter (brown) driven by *FasII-GAL4* (#46123). (**A**) Stage 11 embryo in which the anterior (amg) and posterior (pmg) midgut are stained. (**B**) Stage 13 embryo displaying continuous staining throughout the gut tissue, including the foregut (fg), midgut (mg) and hindgut (hg). (**C, C’**) Early stage 15 embryos in which the esophagus (es) is starting to loop. By this stage the visceral mesoderm that forms the visceral muscles surrounding the gut has migrated to the epithelial lining, but we see no evidence that the *FasII* line expresses in this tissue. (**D**) Late stage 15 embryo with persistent staining throughout the gut. (**E**) Early stage 16 embryo in which the proventriculus (pv) has started to form. Expression is widespread and continues to stage 17/early larval stages but at weaker levels.

We anticipate wanting to be able to manipulate the esophagus and gut tissue over which the SNS precursors migrate and differentiate. We identified a GAL4 line with a fragment of the *FasII* promoter (#46123) that expresses strongly from the earlier stages of gut formation until the point at which SNS precursors stop migrating (Fig 6). The line is striking as it expresses simultaneously in the foregut, midgut and hindgut. As the gut is derived from different cell populations and germ layers [31], GAL4 lines typically express in a subsection of the gut rather than the entire tissue [32]. The *FasII* line identified expresses in the intestinal epithelium as opposed to the visceral muscle that surrounds the gut. Over-expression of the EGFR ligand *Spitz* led to extremely disrupted embryos as would be expected from high expression of a potent growth factor.

## Discussion

### Lines identified in this study

Our analysis has identified three GAL4 lines that have embryonic specific SNS expression with limited expression in other tissues. The *RetP2-GAL4* line expresses in a subset of SNS cells with strong persistent expression in the proventricular ganglion in the first instar larva. The *Gfrl-GAL4* line expresses in most SNS cells until the end of embryogenesis. The *Gsc-GAL4* line expresses in likely all SNS cells, and based on reporter gene expression is the strongest driver we identified in the embryo, although SNS expression does not persist into the larvae. All three lines have utility in SNS manipulation as they produce *EGFR* phenotypes that occur after EGFR’s role in the specification of SNS precursor clusters. As SNS delamination was unaffected by any of the GAL4 lines used, these lines may not be used to manipulate the earliest stages of SNS migration. The strength of reporter genes suggests the GAL4 lines may have relatively low expression. We are currently building stocks to amplify or permanently switch on expression to assess whether the lines will be useful in larval analysis.

### Relationships between identified gene fragments

Striking similarities are observed between the expression patterns of the promoter fragments analyzed. *RetP1-GAL4*, *RetP2A-GAL4*, *RetP2B-GAL4*, *Gfrl-GAL4* (2 different lines), *fkh-GAL4* and *Gsc-GAL4* all express in cells near the back of the brain lobes, in the migrating SNS precursors and/or the underlying esophageal cells, suggesting that these genes may be functionally linked. It has been suggested that *Ret* and *Gfrl* did not function as a *cis* receptor-coreceptor pair before the emergence of GDNF family ligands in vertebrate lineages [25]. The shared expression patterns of *Ret* and *Gfrl* regulatory elements suggest a functional relationship exists in flies and likely other invertebrates. We note that *Ret* and *Gfrl* share expression both in the SNS and in the malphigian tubules [14, 25], the fly homologue of the vertebrate kidney where Ret also plays a role [33, 34]. We are currently generating *Ret* mutants in the fly to establish the precise role it plays in SNS formation.

The *Ret* gene is one of the key markers for neural crest cells migrating into the gut [35] and plays a key role in enteric nervous system formation [12]. Neural crest cells acquire their identities through the expression of neural crest specifier genes such as the Snail, FoxD3 and SoxE genes. The fly Sox10 ortholog is not expressed in the SNS and appears to have been co-opted into neural crest development during the course of evolution through altered expression patterns [36]. It is therefore interesting to note that although the fly FoxD3 homologue is not expressed in the SNS [37], the related *fkh* gene is required for SNS formation [38]. Similarly, *Gsc* homologues have roles in neural crest development [39, 40]. Identifying regulators of SNS development in the fly and their functional relationships has the potential to shed light on vertebrate neural crest formation. Other insects display a similar migration of enteric precursors [41–43], suggesting that SNS precursor migration is an ancient developmental program. It will be interesting to see whether there is an evolutionarily conserved regulatory network driving *Ret* expression. Such information will likely be useful in unraveling the genetic complexity of Hirschsprung’s disease in humans.

### Future directions

Identification of three independent SNS specific drivers with different expression characteristics provides an opportunity to investigate the development of the fly SNS. Relatively few SNS developmental components have been identified and fewer have been characterized to date, particularly in the later stages of embryonic development. Reporter genes can be used as markers to dissect phenotypes in greater detail. The ability to complement loss of function data with gain of function data is an important tool in analyzing function, as is the ability to rescue phenotypes. Some of the lines such as *Ret-P2* may be useful in larval feeding assays that can provide comprehensive functional readouts [44–47]. Hirschsprung’s disease occurs one hundred times more frequently in Down Syndrome patients and overexpression of the Dscam gene is the leading candidate gene [48]. The SNS drivers will facilitate modeling of Hirschsprung's disease in a simple organism.

## Supporting Information

S1 Fig Confocal analysis of EGFR inhibition in the SNS. Anti-Fasciclin II staining (monoclonal antibody 1D4) revealing the mature SNS in lateral views. (A) Wild type (w^1118^) embryo showing the frontal nerve (fn), frontal ganglion (fg), recurrent nerve (rn), supraesophageal or brain commissure (sec), corpora cardiaca (cc), frontal connective (fc), esophageal ganglia (EG), esophageal nerve (en), ventricular ganglion (vg) and ventral nerve cord (vnc). (B) *RetP2A-GAL4* driving the *EGFR* dominant negative transgene. The frontal nerve is absent (arrowheads) and the recurrent nerve is possibly less tightly bundled (arrow). (C) *RetP2B-GAL4* driving the *EGFR* dominant negative transgene. The frontal nerve is absent (arrowheads) whereas the recurrent nerve looks normal.
(TIF)

S2 Fig SNS manipulation using the *GscG-GAL4* line. Anti-Fasciclin II staining (monoclonal antibody 1D4) revealing the mature SNS in ventral views. (**A**) Stage 17 wild type embryo with elements of the SNS labeled. The recurrent nerve (rn, arrow) runs from the esophageal ganglion (EG) along the esophagus underneath the brain commissure (bc) to the esophageal gangion (EG). (**B**) *GscG-GAL4* driving transgenic RNAi for *EGFR*. The recurrent nerve (arrow) is clearly disrupted. (**C**) *GscG-GAL4* driving an EGFR dominant negative transgene. The frontal nerve is missing (arrowhead) and the recurrent nerve is disrupted.
(TIF)
