# Supplementary figures and images for: Genetic Tools for the Analysis of Drosophila Stomatogastric Nervous System Development
Source: PLoS One. 2015 Jun 8;10(6):e0128290. doi: 10.1371/journal.pone.0128290 (PMC4460011; doi:10.1371/journal.pone.0128290)

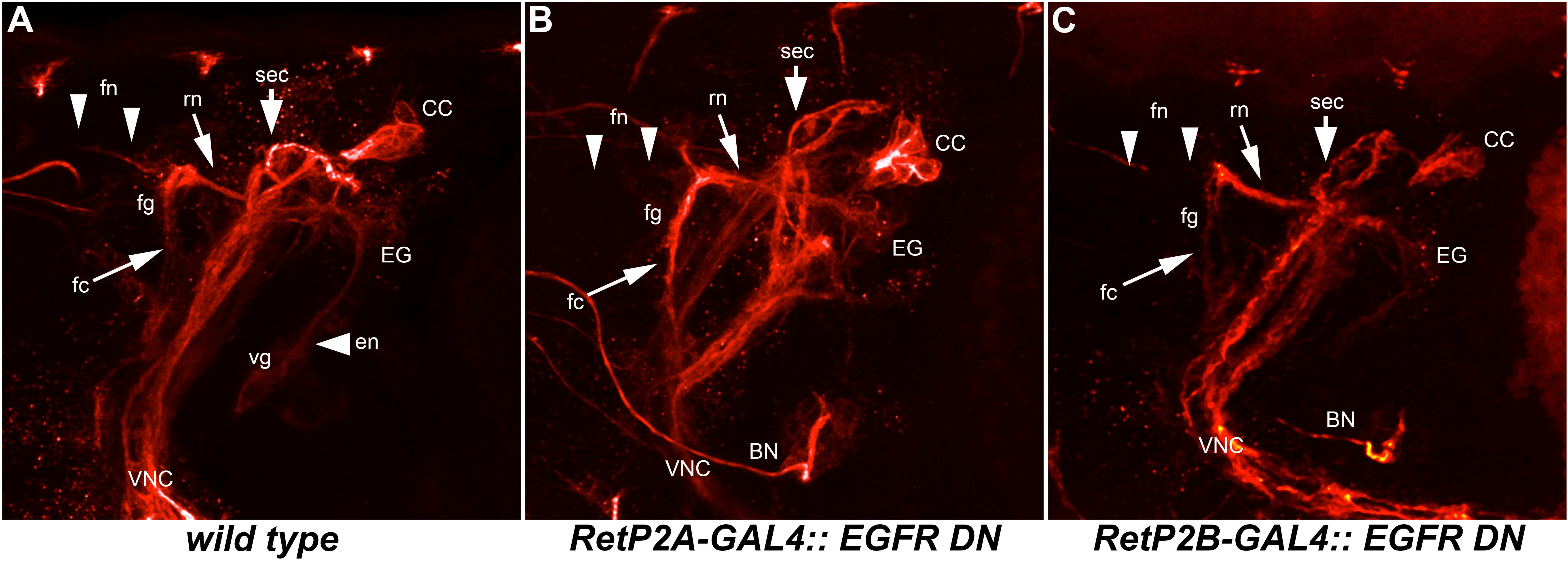

Supplement: S1 Fig — Anti-Fasciclin II staining (monoclonal antibody 1D4) revealing the mature SNS in lateral views. (A) Wild type (w1118) embryo showing the frontal nerve (fn), frontal ganglion (fg), recurrent nerve (rn), supraesophageal or brain commissure (sec), corpora cardiaca (cc), frontal connective (fc), esophageal ganglia (EG), esophageal nerve (en), ventricular ganglion (vg) and ventral nerve cord (vnc). (B) RetP2A-GAL4 driving the EGFR dominant negative transgene. The frontal nerve is absent (arrowheads) and the recurrent nerve is possibly less tightly bundled (arrow). (C) RetP2B-GAL4 driving the EGFR dominant negative transgene. The frontal nerve is absent (arrowheads) whereas the recurrent nerve looks normal. (TIF) [file pone.0128290.s001.tif]

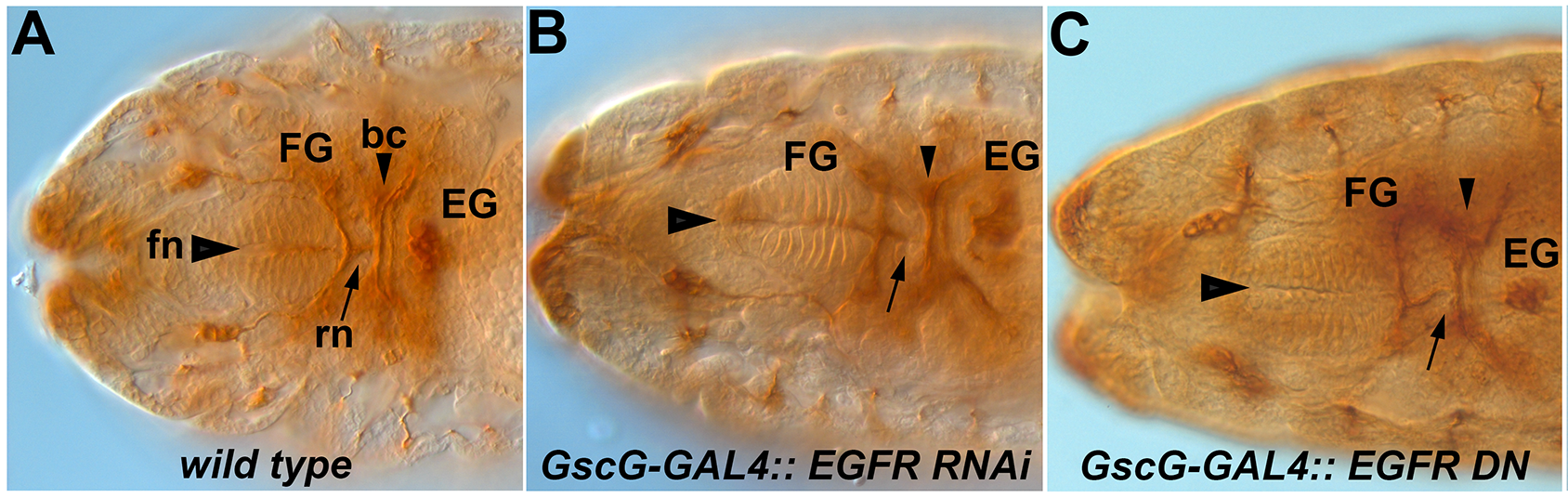

Supplement: S2 Fig — Anti-Fasciclin II staining (monoclonal antibody 1D4) revealing the mature SNS in ventral views. (A) Stage 17 wild type embryo with elements of the SNS labeled. The recurrent nerve (rn, arrow) runs from the esophageal ganglion (EG) along the esophagus underneath the brain commissure (bc) to the esophageal gangion (EG). (B) GscG-GAL4 driving transgenic RNAi for EGFR. The recurrent nerve (arrow) is clearly disrupted. (C) GscG-GAL4 driving an EGFR dominant negative transgene. The frontal nerve is missing (arrowhead) and the recurrent nerve is disrupted. (TIF) [file pone.0128290.s002.tif]
